# Estimation and Characterization of Dengue Serotypes in Patients Presenting with Dengue Fever at Makkah Hospitals

**DOI:** 10.3390/tropicalmed10010027

**Published:** 2025-01-20

**Authors:** Sami Melebari, Abdul Hafiz, Hatim A. Natto, Mohamed Osman Elamin, Naif A. Jalal, Ashwaq Hakim, Safiah Rushan, Othman Fallatah, Kamal Alzabeedi, Feras Malibari, Hutaf Mashat, Aisha Alsaadi, Amani Alhakam, Anoud Hadidi, Ghazi Saad Alkhaldi, Ahmed Alkhyami, Ali Alqarni, Abdulaziz Alzahrani, Mohammed Alghamdi, Abdullah Siddiqi, Abdullah Alasmari, Rowaida Bakri, Saleh Alqahtani, Juman M. Al-Bajaly, Asim Khogeer

**Affiliations:** 1 Department of Molecular Biology, The Regional Laboratory, Ministry of Health, Makkah 21955, Saudi Arabia; samelibari@yahoo.com (S.M.); athakim@moh.gov.sa (A.H.); hmmashat@moh.gov.sa (H.M.); aialsaadi@moh.gov.sa (A.A.); aalhakam@moh.gov.sa (A.A.); ahadidi@moh.gov.sa (A.H.); 2 Department of Microbiology and Parasitology, Faculty of Medicine, Umm Al-Qura University, Makkah 21955, Saudi Arabia; najalal@uqu.edu.sa (N.A.J.); rabakari@uqu.edu.sa (R.B.); 3 Department of Epidemiology and Biostatistics, Faculty of Public Health & Informatics, Umm Al-Qura University, Makkah 21955, Saudi Arabia; hanatto@uqu.edu.sa; 4 Department of Environmental and Occupational Health, Faculty of Public Health and Informatics, Umm Al-Qura University, Makkah 21955, Saudi Arabia; mobushara@uqu.edu.sa; 5 Department of Serology, The Regional Laboratory, Ministry of Health, Makkah 21955, Saudi Arabia; srushan@moh.gov.sa (S.R.); ofallatah@moh.gov.sa (O.F.); aalkhyami@moh.gov.sa (A.A.); aaalgarni@moh.gov.sa (A.A.); aalzahrani737@moh.gov.sa (A.A.); moabal-ghamdi@moh.gov.sa (M.A.); 6 Departments of Medical Research, Clinical Biochemistry, The Regional Laboratory, Ministry of Health, Makkah 21955, Saudi Arabia; kalzabeedi@moh.gov.sa; 7 Epidemiology and Infection Control, Saudi German Hospital, Makkah 21955, Saudi Arabia; epi.feras@gmail.com; 8 Department of Immunology, The Regional Laboratory, Ministry of Health, Makkah 21955, Saudi Arabia; gsalkhaldi@moh.gov.sa; 9 Department of Clinical Laboratory, Makkah Park Clinics, Makkah 21955, Saudi Arabia; a.siddiqi@makkahparkclinics.sa; 10 Department of Research, PMO, Ministry Branch in Makkah Region, Ministry of Health (MOH), Makkah 21955, Saudi Arabia; aal-asmri@moh.gov.sa (A.A.); sal-qahtani@moh.gov.sa (S.A.); jalbajaly@moh.gov.sa (J.M.A.-B.); akhogeer@moh.gov.sa (A.K.); 11 Medical Genetics Unit, Maternity & Children Hospital, Makkah Healthcare Cluster, Ministry of Health (MOH), Makkah 21955, Saudi Arabia

**Keywords:** dengue, Ades, DENV, travel

## Abstract

Dengue fever is caused by four common serotypes of the dengue virus (DENV-1, DENV-2, DENV-3, and DENV-4). Patients infected with one serotype may develop lifelong serotype-specific protective immunity. However, they remain susceptible to reinfection with the other serotypes, often increasing the risk of severe forms of dengue. This cross-sectional study investigates the prevalence of the four dengue serotypes in patients who presented with dengue fever at Makkah hospitals between April 2023 and May 2024. Data were collected from the medical records of the Regional Laboratory in Makkah, Saudi Arabia. The 238 positive dengue samples included 185 samples (77.73%) from male patients. The average age of the patients was 37.65 years (SD = 15.05). Dengue type 2 was the most common serotype, followed by type 1, type 3, and type 4. Most of the dengue patients were Saudi nationals, followed by Egyptians. There were 11 dengue-positive samples that were not diagnosed with any of the four dengue serotypes. Since Makkah receives numerous international travelers, these samples might contain novel dengue serotypes circulating in different parts of the world. This study underscores the need for the continuous monitoring of dengue serotypes to predict potential outbreaks and mitigate the risk of severe dengue in susceptible populations.

## 1. Introduction

Dengue is a mosquito-borne viral disease caused by the dengue virus (DENV) that has enormous public health significance. DENVs belong to the Flaviviridae family of single-stranded RNA viruses that have a positive genetic strand. Almost half of the world’s population lives in areas at risk of dengue infection. An estimated 100–400 million people are infected with the dengue virus annually [1]. However, only a few of these infections develop into clinical cases. In 2023, there were an estimated 6.5 million cases of dengue, and it caused 6800 deaths globally [2]. In the WHO East Mediterranean Region, dengue is endemic in Afghanistan, Djibouti, Egypt, Oman, Pakistan, Saudi Arabia, Somalia, Sudan, and Yemen. Two vector species are involved in DENV transmission in this region: *Ae. aegypti*, which is the primary vector, and *Ae. albopictus*, which is considered to be the secondary vector [3].

Dengue is more common in tropical and subtropical regions. However, due to environmental changes and increased global travel and transportation, mosquito vectors have established themselves in newer locations, and thus has the dengue virus [4]. Most DENV infections may be subclinical or asymptomatic and thus remain undiagnosed. In the majority of dengue cases, the illness first causes a moderate fever, but after three to fifteen days, the infected person may experience various symptoms, including a very high temperature [5,6]. However, a small percentage of dengue infections may turn into severe, life-threatening conditions. The progression of dengue disease from non-severe to severe forms, such as Dengue Hemorrhagic Fever (DHF) or Dengue Shock Syndrome (DSS), is influenced by multiple factors. Understanding these factors is crucial for predicting and managing severe dengue cases [7,8,9]. Key factors include the infecting viral serotype, host immune history and status, environmental conditions, and the timing of medical intervention or access to healthcare.

Dengue virus infections elicit serotype-specific host immune responses [10,11]. There are four distinct serotypes of the dengue virus, known as DENV-1, DENV-2, DENV-3, and DENV-4, and they are detected quite frequently [12]. However, a rare fifth serotype has also been reported [13]. The distinct dengue virus serotypes share over 65% of their genomic identity and are genetically related [14,15,16], meaning they differ in their antigenic properties but are closely related enough to cause similar clinical symptoms. While infection with any one serotype typically provides long-lasting immunity against that specific serotype, it may not help elicit a protective immune response against the other serotypes. In fact, subsequent infection with a different serotype can lead to more severe forms of the disease [17]. DENV-2 is frequently linked to more serious outcomes, especially in secondary infections. Several factors contribute to the severity of DENV-2, including antibody-dependent enhancement of the infection, making DENV-2 one of the most virulent serotypes from a public health perspective [17,18,19].

In addition, DENV-5, a new serotype of the dengue virus, was reported in 2013 [20,21]. However, this new serotype has not yet been approved by the International Committee on Taxonomy of Viruses (ICTV). The discovery of DENV-5 highlights the complexity of dengue virus transmission and evolution. While this serotype has not yet caused widespread human outbreaks, its presence underscores the need for continued surveillance and research to better understand its potential impact on public health [21].

Dengue is considered an endemic disease in certain regions of Saudi Arabia, such as the western and southwestern coastal areas, including the cities of Jeddah, Makkah, and Medina. These regions have favorable climates for the mosquito *Aedes aegypti*, which is the primary vector for the dengue virus in the area [22,23,24,25]. The Saudi Arabian government, through the Ministry of Health (MOH), has implemented control measures to combat dengue, including mosquito control programs, public health campaigns, and surveillance systems [26]. Despite these efforts, dengue outbreaks still occur in rainy seasons as well as sporadically [27]. In addition, the city of Makkah receives millions of visitors from different parts of the world, including areas that are dengue endemic zones, to perform prayers and undertake *Umrah* and *Hajj*. It is important to monitor the dengue serotypes circulating in this city to help dengue control efforts in Makkah as well as in Saudi Arabia in general [28].

The purpose of this study is to examine reported cases of dengue in Makkah; determine the age, gender, and nationality distribution of these dengue cases; and identify the dengue serotypes present in the city.

## 2. Materials and Methods

### 2.1. Study Site and Population

Makkah is a city of religious importance in Islam located in Saudi Arabia. *Al-Masjid Al-Haram*, the most important *Masjid* in Islam, is situated in Makkah. Millions of Muslims travel to Makkah from different parts of the world every year. It is Saudi Arabia’s third most populous metropolis. In 2023, Makkah’s population was 2 million. The population fluctuates significantly during the *Hijri* months of *Ramadan* and the annual *Hajj* pilgrimage as millions of Muslims from around the world travel to Makkah. During the month of *Ramadan* and the *Hajj* pilgrimage, the population of this city can swell, increasing by a few million additional people, reflecting the massive influx of pilgrims [29]. Makkah’s climate is hot in the summer, often being more than 40 °C during the day and dipping to 30 °C at night. Nonetheless, it is mild during the winter, with daytime highs of 30 °C and nighttime lows of 18 °C. Makkah often receives a few inches of rain between November and January which may cause fluctuations in the dengue-vector population [28,30,31].

### 2.2. Study Design

This is a cross-sectional data analysis study. We enrolled laboratory-confirmed dengue patients from Makkah hospitals between April 2023 and May 2024. Patients with negative dengue diagnoses in the laboratory were excluded. The dengue case definition used in this study was in accordance with the Ministry of Health, Saudi Arabia [32,33]. This study’s participants were Makkah city residents, citizens, and travelers/visitors who had been diagnosed with dengue at Makkah hospitals. Demographic details, including age, gender, and nationality, were collected from the healthcare-providing hospitals.

### 2.3. Criteria for Suspected and Confirmed Dengue Cases

A suspected dengue case was defined as a person with acute febrile illness or a history of acute febrile illness for the past 2–7 days, plus two or more of the additional dengue symptoms as determined by a physician. These additional symptoms include headache, arthralgia, retro-orbital pain, myalgia, rash, leucopenia, and hemorrhagic manifestations [34]. A confirmed dengue case was defined as a person whose sample was identified as positive via quantitative reverse transcription polymerase chain reaction (RT-qPCR) during dengue diagnosis.

### 2.4. Collecting Samples

Samples from all suspected dengue cases were collected from Makkah hospitals and were forwarded to the regional laboratory in Makkah to test for anti-dengue immunoglobulins (IgMs) using an enzyme-linked immunoassay (ELISA) and the dengue virus RNA via polymerase chain reaction (RT-PCR), as described in the protocol [34]. The results were recorded on the HESN plus webpage. The Saudi Arabian Ministry of Health (MOH) requires all its laboratories to record laboratory results on this webpage. The HESN is part of MOH’s Epidemiological Surveillance Program [35]. For further molecular studies, the materials were maintained at −80 °C in constant freezing conditions.

### 2.5. Extraction of Ribonucleic Acid (RNA)

RNA was extracted from 300 μL of a serum specimen utilizing a MagNA Pure 96 Instrument (Roche Molecular Systems, Inc., Mannheim, Germany) according to the instructions provided by the manufacturer.

### 2.6. Quantitative Reverse Transcription Polymerase Chain Reaction (RT-qPCR) and Reverse Transcription Polymerase Chain Reaction (RT-PCR)

The RT-qPCR tests were performed using a LightMix Modular Dengue Virus kit (Roche Diagnostics (Schweiz) AG), as per the instructions given by the manufacturer. Precisely 10 μL of the reaction mixture was added to 10 μL of the purified RNA. The PCR reaction per well included 4.0 μL of Roche master; 0.1 μL of the RT enzyme; 0.5 μL of the reagent mix, including primers and probes; and 5.4 μL of PCR-grade water. The reverse transcription phase was completed using the following parameters: a 55 °C RT step for 5 min, and then 95 °C denaturation for 5 min, followed by 45 cycles of 5 s at 95 °C, 15 s at 60 °C, and 15 s at 72 °C in quantification mode. Finally, the reaction was cooled at 40 °C for 30 s. All procedures were performed in a LightCycler 480 instrument, and results were analyzed using the LightCycler software 480 (Roche Molecular Systems, Inc., Germany). RT-PCR was used for serotyping. The procedure was similar to RT-qPCR except that a Realstar^®^ Dengue Type RT-PCR Kit 1.0 from Altona Diagnostics was used, as per the instructions supplied with the kit. The kit does not use any housekeeping gene probe for internal control; instead, it uses a heterologous amplification system as an internal control to identify possible RT-PCR inhibition and confirm the integrity of the reagents of the kit. Since it uses a “heterologous amplification system” instead of a housekeeping gene as an internal control, this kit cannot determine the integrity of RNA obtained from a patient. It can only determine the integrity of the reagents of the kit and the inhibition of the RT-PCR reaction.

### 2.7. Enzyme-Linked Immunosorbent Assay (ELISA)

The ELISA method was utilized to qualitatively detect the NS1 antigen (Platelia^TM^ Dengue NS1 Ag, Bio-RAD, Marnes-la-Coquette, France) and IgG and IgM human antibodies against DENV (SERION ELISA classic, Dengue Virus IgG/IgM). All the internal controls and samples given in each kit were treated according to the manufacturer’s instructions, using a completely automated ELISA system. (Human Diagnostics, Wiesbaden, Germany).

### 2.8. Statistical Analysis

Data were input into Microsoft Excel spreadsheets. The data inputs were double-checked to eliminate any data entry errors. All data analysis and curve plotting for data display were performed using Microsoft Excel.

## 3. Results

A total of 238 positive dengue samples were studied from April 2023 to May 2024. Of these samples, 77.73% (n = 185) and 22.27% (n = 53) were from male and female patients, respectively. The average age of the patients was 37.65 years (standard deviation = 15.05). The average Ct value for RT-qPCR was 27.33 (standard deviation = 4.35). Serological tests revealed that 81.51% of the analyzed samples were positive for the NS1 antigen ELISA (n = 194), 78.57% were positive for the IgM antibody ELISA (n = 187), and 44.53% were positive for the IgG antibody ELISA (n = 106) (Table 1). There were differences in the serological findings and RT-qPCR Ct values between male and female patients (Table 1). Male patients were greater in number than female patients in all but three hospitals (Table A1). SUHAIL hospital, which caters to patients coming for *Hajj* and *Umrah* near *Al-Masjid Al-Haram*, reported the highest number of male and female patients (Table A1).

**Figure 1:**
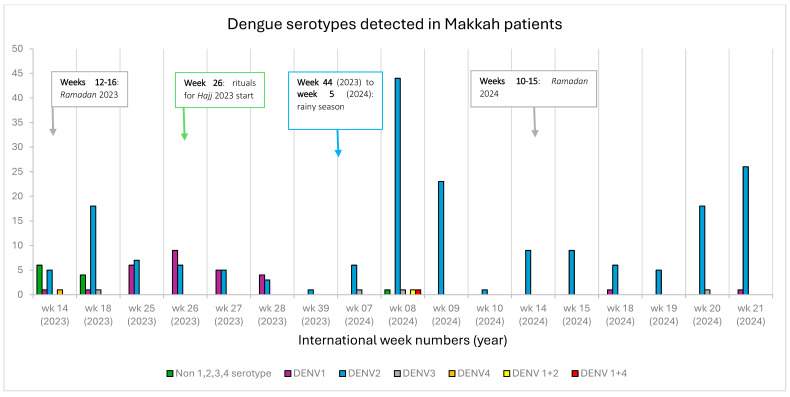
Dengue serotypes detected in different weeks in 2023 and 2024. The different colored bars show the number of various dengue serotypes detected according to international weeks in 2023 and 2024. The weeks pertaining to the month of *Ramadan* in 2023 (22 March–20 April), the month of *Ramadan* in 2024 (10 March–09 April), the start date of *Hajj* rituals (26 June 2023), and the rainy season in Makkah (November–January) are marked with different colored arrows.

The study period comprised sixty weeks (fourteen months). However, dengue cases were observed only for a total of seventeen weeks. The DENV2 serotype was detected throughout these seventeen weeks, while DENV1 was found in weeks 14, 18, 25, 26, 27, and 28 of 2023 and weeks 18 and 21 of 2024. Most of the non 1,2,3,4 serotype samples were diagnosed in weeks 14 and 18 of 2023 (Figure 1).

Patients of several nationalities were found to be dengue-positive (Table 2). Saudi patients accounted for 40.34% of the total (n = 96) followed by 18.07% (n = 43) patients from Egypt, 9.24% (n = 22) from Pakistan, and 5.88% (n = 14) of unknown nationalities. Bangladesh and India each accounted for 5.04% (n = 12) of confirmed cases. Yemen accounted for 4.62% (n = 11) of the total dengue cases. Myanmar and Sudan accounted for 2.94% (n = 7) of the patients. Afghanistan, Algeria, and Iraq contributed to 1.26% (n = 3) of cases. Syria accounted for 0.84% of cases (n = 2) while Mali, Nepal, and Tunisia accounted for 0.42% of the cases (n = 1).

An age distribution analysis was conducted to determine the distribution of dengue infection by age (Table 3). The 21–30-year-old and 31–40-year-old age groups each accounted for 26.89% of the total dengue cases (n = 64, each). The 41–50 age group accounted for 15.97% of cases (n = 38), followed by the 51–60 age group, which accounted for 12.18% (n = 29).

All four main dengue serotypes reported in Saudi Arabia were also detected in our patients. Dengue type 2 was detected in 80.25% of patients (n = 191), followed by serotype 1 in 12.18% (n = 29), serotype 3 in 1.68% (n = 4), and serotype 4 in 0.42% (n = 1) of our study patients. However, about 4.62% (n = 11) of the patients were infected with a dengue serotype that could not be identified in our assays. In these 11 samples, 81.81% were reactive to NS1 antigen, 72.72% were reactive to IgM, and 54.54% were reactive to IgG antibodies in serological dengue diagnostic tests. The reactivity in serological tests for NS1 and IgM, the gender distribution, the average ages, and the average Ct values for these 11 samples were similar to those of the total study samples. However, reactivity to IgG was higher in these 11 samples compared to the total samples (54.54% vs. 44.53%) (Table 1).

## 4. Discussion

The city of Makkah in the Kingdom of Saudi Arabia receives millions of local and international travelers every year. Makkah has a population of 2 million. It received almost 1.8 million *Hajj* pilgrims in 2024. More than 10 million *Umrah* performers visited *Al-Masjid Al-Haram* in the *Hijri* month of *Ramadan* in 2022 [29]. In addition, millions of foreigners live in the Kingdom as residents. Infectious diseases come with the local and international travelers to Makkah. These travelers come from almost every part of the world. The city of Makkah has been receiving international travelers since ancient times, impacting almost all aspects of human life including public health. In the past, the influx of foreign religious travelers was limited, as international travel was not as fast and convenient as it is these days. In modern times, the number of *Umrah* and *Hajj* travelers has considerably increased.

The bulk of travelers to Makkah come in the *Hijri* months of *Ramadan* and *Dhul-Hijjah* (the month of *Hajj*). These visitors may come to Makkah a few weeks before these months, and some stay a few weeks after the end of this period. It is quite interesting to note that the non-1, -2, -3, or -4 serotypes and DENV1 were diagnosed in and around the months of *Ramadan* and *Dhul Hijjah*. No dengue cases were diagnosed during the annual rainy season from November to January. However, the highest number of dengue-positive samples in Makkah were diagnosed two weeks after the end of the rainy season, implicating rain in the transmission of this mosquito-borne disease. Vector-born disease transmission patterns are influenced by several environmental factors, including rain, changes in land use, human movements, and so on [36,37].

Our study suggests that most of the dengue patients were Saudi nationals who could have been Makkah residents or had come to Makkah from different parts of the country. Dengue is endemic in Saudi Arabia [24]. The infections were more common in adult males between 21 and 30 and 31 and 40 years old.

More male than female patients were diagnosed with dengue in Makkah during the study period. This phenomenon has been reported in several previous studies [36,37]. Moreover, dengue was less prevalent in children and the elderly. Females, children, and the elderly may not be exposed to mosquito bites in the same proportion as adult males who are regularly engaged in fieldwork. In Makkah, foreign men come more frequently than women for *Umrah*, *Hajj*, and work. Cleaners, construction workers, security personnel, and vehicle drivers that transport visitors and goods from other cities to Makkah are also mostly male. Studies characterizing the professions and socio-economic statuses of dengue patients may provide interesting insights into dengue transmission patterns.

A comparison between the two gender groups in our study shows that women had higher Ct values, higher proportions of IgG-reactive samples, and lower proportions of NS1- and IgM-reactive samples than men. This suggests that women might have been diagnosed with dengue infection later as compared to men in our study. There might be differences in the health-seeking behaviors of the two genders. Such gender-based differences in the number of reported dengue cases are consistently observed in multiple culturally and economically different countries [38,39]. It should be noted that only an estimated 1–6% of dengue infections develop into clinical cases [2]. It would be interesting to determine if there are gender-associated determinants of dengue disease manifestation or severity. The gender differences observed in this study warrant further investigation into potential behavioral or biological factors.

Interestingly, 11 of our dengue-positive samples did not react to type-1-, -2-, -3-, or -4-specific RT-PCR assays. The general and laboratory characteristics of these 11 samples were comparable to those of the total study samples, suggesting there are no other anomalies in the laboratory findings for these samples. The average Ct value of the RT-qPCR was also comparable with that of the total samples. However, the possibility of template degradation or the presence of PCR inhibitors in the samples during serotyping RT-PCR remains. Since Makkah receives millions of global visitors, the presence of novel or rare dengue serotypes cannot be ruled out either. Further studies with molecular and viral genome-sequencing approaches are needed to determine the presence of novel dengue serotypes.

The study had several limitations; therefore, the results should be interpreted with caution. Firstly, we recruited only suspected dengue patients presenting at various hospitals in Makkah. Therefore, this study does not reflect on the total number of dengue infections in Makkah. Secondly, not all dengue cases diagnosed among foreigners are traveler’s infections. Several foreigners live in Saudi Arabia for work and business as residents. Finally, people may have altered health-seeking behavior when they travel as compared to when they are at the comfort of their homes. Similarly, there may be differences in health-seeking behavior among people of different genders, age groups, social as well as economic groups.

## 5. Conclusions

Dengue is endemic in Saudi Arabia. We observed 238 dengue cases during the study period. Most of the dengue patients in our study were Saudi nationals. In addition to the prompt diagnosis and treatment of dengue cases, continuous case surveillance and vector control efforts are important to contain the disease in Makkah. Dengue serotype 2 was the most prevalent serotype in Makkah during the study period. Dengue was more frequently diagnosed in males than females. It would be quite interesting to determine the underlying causes for this observed gender-based difference. We could not determine the dengue serotypes in the samples collected from 11 patients. Further research is needed to confirm the presence of uncommon or rare dengue serotypes in Makkah.

## Figures and Tables

**Table 1:** The general and laboratory characteristics of the study samples.

	All Samples (n = 238)	Non 1,2,3,4 Serotype (n = 11)	Samples from Male Patients (n = 185)	Samples from Female Patients (n = 53)
**Average age (St Dev)**	37.65 (15.05)	33.9 (14.63)	36.83 (13.89)	40.49 (18.12)
**Gender (male patients)**	77.73%	72.72%	100%	0%
**NS1 reactive**	81.51%	81.81%	83.78%	73.58%
**IgM reactive**	78.57%	72.72%	80.54%	71.69%
**IgG reactive**	44.53%	54.54%	43.78%	47.16%
**Average Ct (St Dev)**	27.33 (4.35)	26.21 (8.00)	26.85 (3.99)	28.99 (5.16)

**Table 2:** Dengue serotypes and nationalities of the patients. Dengue serotype 2 was the most common, followed by type 1. There were 11 samples that did not react to the detection kits for types 1, 2, 3, or 4 (non 1, 2, 3, and 4 type). Two cases of mixed serotype infections were also detected.

Nationality	Dengue Type							Total	% of Total
	Type 1	Type 2	Type 3	Type 4	Non 1,2,3,4	Type 1 + 2	Type 1 + 4		
**Saudi Arabia**	**13**	75	2	1	4	1	-	96	40.34
**Egypt**	2	38	1		2	-	-	43	18.07
**Pakistan**	-	18	1		3	-	-	22	9.24
**Unknown**	1	12	-	-	-	-	1	14	5.88
**Bangladesh**	-	12	-	-	-	-	-	12	5.04
**India**	4	8	-	-	-	-	-	12	5.04
**Yemen**	2	8	-	-	1	-		11	4.62
**Myanmar**	-	7	-	-	-	-	-	7	2.94
**Sudan**	2	4	-	-	1	-	-	7	2.94
**Afghanistan**	1	2	-	-	-	-	-	3	1.26
**Algeria**	2	1	-	-	-	-	-	3	1.26
**Iraq**	2	1	-	-	-	-	-	3	1.26
**Syria**	-	2	-	-	-	-	-	2	0.84
**Mali**	-	1	-	-	-	-	-	1	0.42
**Nepal**	-	1	-	-	-	-	-	1	0.42
**Tunisia**	-	1	-	-	-	-	-	1	0.42
**Total**	29	191	4	1	11	1	1	238	100.00

**Table 3:** Dengue cases arranged by age group. Mixed infections were only observed in the 41–50 age group. The non 1, 2, 3, 4 serotype had a broad distribution in all age groups, except for those 11–20 years of age and 70+ years old.

Age Groups	Dengue Type							Total	% of Total
	Type 1	Type 2	Type 3	Type 4	Non 1,2,3,4	Type 1 + 2	Type 1 + 4		
**0–10 years**	**-**	4	-	-	1	-	-	5	2.10
**11–20 years**	4	12	1	-	-	-	-	17	7.14
**21–30 years**	6	52	2	-	4	-	-	64	26.89
**31–40 years**	3	59	-	1	1	-	-	64	26.89
**41–50 years**	6	27	-		3	1	1	38	15.97
**51–60 years**	6	21	1	-	1	-	-	29	12.18
**61–70 years**	1	12	-	-	1	-	-	14	5.88
**70+ years**	2	5	-	-	-	-	-	7	2.94
**Total**	28	192	4	1	11	1	1	238	100.00

## Data Availability

Data can be obtained from the corresponding author after approval from the Ministry of Health, Saudi Arabia.

## Appendix A

**Table A1:** Dengue cases arranged according to the referring hospital. The number of male and female cases is listed by the name of the referring hospital in Makkah.

Hospital Name	Number of Males	Number of Females
ALAHLI	11	2
ALABEER	1	1
ALBADR	1	-
ALBEYAT MEDICAL	11	-
ALHUDA	3	-
ALRAFIE	1	-
ALSHEFA	1	1
BASHARAHEEL	4	1
HERA	8	8
KING ABDULAZIZ	8	2
KING FISAL	17	8
KHULAIS	2	2
MAKKAH MEDICAL	21	4
MATERNITY&CHILDREN	1	2
ALNOOR	26	3
SAUDI HOSPITAL	5	-
SECURITY FORCES	8	5
SUHAIL	56	10
KING ABDULAZIZ MEDICAL CENTRE	-	3
SAUDI GERMAN	-	1
**Total**	**185**	**53**
